# The relationship between 5α-reductase inhibitors and the presence of following open angle glaucoma: a TriNetX cohort study

**DOI:** 10.7150/ijms.137426

**Published:** 2026-10-02

**Authors:** Chia-Yi Lee, Shun-Fa Yang, Shih-Chi Su, Yu-Ling Chang, Jing-Yang Huang, Chao-Bin Yeh, Po-Hsien Huang, Chao-Kai Chang

**Affiliations:** 1 Institute of Medicine, Chung Shan Medical University, Taichung, Taiwan.; 2 Cerulean Eye Center, Taichung, Taiwan.; 3 Nobel Eye Institute, Taipei, Taiwan.; 4 Department of Medical Research, Chung Shan Medical University Hospital, Taichung, Taiwan.; 5 Whole-Genome Research Core Laboratory of Human Diseases, Chang Gung Memorial Hospital, Keelung, Taiwan.; 6 Department of Pediatrics, Cathay General Hospital, Taipei, Taiwan.; 7 Department of Emergency Medicine, School of Medicine, Chung Shan Medical University, Taichung, Taiwan.; 8 Department of Emergency Medicine, Chung Shan Medical University Hospital, Taichung, Taiwan.; 9 Kaohsiung Nobel Eye Institute, Kaohsiung, Taiwan.; 10 Graduate Institute of Clinical Medicine, Kaohsiung Medical University, Kaohsiung, Taiwan.

**Keywords:** epidemiology, TriNetX database, finasteride, 5α-reductase inhibitors, open angle glaucoma

## Abstract

**Purpose:**

This study was conducted to test a possible association between 5α-reductase inhibitor (5ARI) use and subsequent open-angle glaucoma (OAG).

**Method:**

A retrospective cohort study was conducted, and individuals with benign prostate hyperplasia (BPH) and androgenetic alopecia were included and classified according to whether they received 5ARI treatment. A total of 181,208 individuals were included in each of the 5ARI and non-5ARI groups. The primary outcome was the development of OAG according to diagnostic and procedure codes. Cox proportional hazard regression was performed for the statistical analysis.

**Results:**

OAG occurred in 3477 patients (5.14%) in the 5ARI group and 3044 patients (4.59%) in the non-5ARI group. After adjustment for multiple covariates, exposure to 5ARIs was associated with a significantly greater risk of OAG compared with the non-5ARI treatment (P < 0.001). The cumulative incidence of OAG was significantly higher among patients receiving 5ARI therapy than among those in the non-5ARI group (P < 0.001), and the cumulative incidence of HTG was significantly higher in the 5ARI population than the non-5ARI population (P = 0.002). In the sensitivity analysis, the 5ARI cohort exhibited significantly increased rates of OAG and HTG compared with the non-5ARI cohort, except for Asian individuals (P > 0.05).

**Conclusions:**

The use of 5ARIs may be related to the development of OAG.

## Introduction

Hormones play a crucial role in regulating human metabolism, tissue remodeling, and reproductive physiology 1. Among the various endocrine mediators of the human body, androgens are particularly important for the development of both male reproductive organs and secondary sexual characteristics 2. In addition to their physiological significance, androgens may also participate in the pathogenesis of certain disorders, including benign prostatic hyperplasia (BPH) and androgenetic alopecia 3-5. To manage these androgen-dependent disorders, 5α-reductase inhibitors (5ARIs) have been used for many years, and previous studies have generally reported satisfactory therapeutic efficacy for this approach 6, 7.

Although 5ARI therapy can effectively treat androgen-associated diseases, its use has also been linked to several adverse events and related comorbidities 8, 9. Earlier research suggested that exposure to 5ARIs may increase the likelihood of insulin resistance and type 2 diabetes mellitus 9. Sexual and reproductive complications have also been described, including a greater occurrence of erectile dysfunction and impaired fertility among patients receiving 5ARI treatment 10. In addition, another publication reported a possible association between the administration of 5ARIs and an elevated risk of depressive disorders 11. Furthermore, a recent review article indicated that 5ARI use may be associated with the development of bladder and kidney cancers 12.

Open-angle glaucoma (OAG) is an ocular disease characterized by visual field defects and can be classified as either high-tension glaucoma (HTG) or normal-tension glaucoma (NTG) 13, 14. In previous research, the development of OAG has been linked to other medications, including corticosteroids and calcium channel blockers 15. However, few studies have evaluated the potential association between OAG and 5ARI use. Because 5ARIs may interfere with the vasculature and vascular defects play a critical role in OAG development 16, 17, such an association may exist, thus requiring evaluation.

As a result, the objective of this study is to investigate the association between 5ARI use and succeeding OAG episodes. In addition, OAG was divided into HTG and NTG events, and their associations to the 5ARI treatment were analyzed separately.

## Materials and Methods

### Data Source

This study was conducted in accordance with the principles of the Declaration of Helsinki of 1964 and its subsequent revisions. The Institutional Review Board of Chung Shan Medical University (project code: CS2-23180) approved this research. Data were retrieved from the TriNetX database, a worldwide federated health research network that provides access to electronic medical records from major healthcare organizations, including diagnostic information, laboratory results, procedures, genomic data, and medication records. Analysis was performed using the US Collaborative Network, which includes data from 108 healthcare institutions. TriNetX collects de-identified electronic health information from participating healthcare systems, most of which are large academic medical centers providing both outpatient and inpatient services in different regions of the United States. Through a secure web-based interface, TriNetX Analytics enables access to patient-level electronic health data from primary care facilities, hospitals, and specialty care providers. As a result, the TriNetX database includes patients from varied geographic locations, age groups, racial and ethnic backgrounds, income levels, and insurance categories, including commercial insurance, Medicare, Medicaid, workers' compensation, self-pay, uninsured status, military insurance, and veterans' affairs coverage. The clinical information available in TriNetX includes International Classification of Diseases, Tenth Revision, Clinical Modification (ICD-10-CM) codes; sex; age; employment status; residential information; educational level; socioeconomic and psychosocial factors; hospitalization duration, if available; imaging, laboratory test, surgical, and procedure codes; and Anatomical Therapeutic Chemical (ATC) codes for medications.

### Patient Selection

A retrospective cohort design was utilized with data from the TriNetX resource. Eligible patients were selected according to the following criteria: (1) diagnosis of androgenetic alopecia or benign prostatic hyperplasia (BPH) based on relevant ICD-10-CM codes, (2) age greater than 50 years according to demographic records, (3) male sex, and (4) diagnosis of androgenetic alopecia or BPH between January 1, 2015 and December 31, 2024. The index date was defined as three months after the diagnosis of androgenetic alopecia or BPH. To improve the consistency of the baseline ocular status among included individuals, several exclusion criteria were applied. Patients were excluded if they had (1) blindness before the index date according to relevant ICD-10-CM codes, (2) ocular malignancy before the index date, (3) a history of eyeball removal surgery before the index date according to surgical codes, (4) severe ocular trauma before the index date, (5) 5ARI treatment exposure before 2015, or (6) the study outcome developed before the index date. Participants were subsequently divided into two cohorts. The 5ARI group consisted of patients who received 5ARI therapy based on ATC codes, whereas the non-5ARI group included patients treated with minoxidil, alpha blockers, or transurethral resection of the prostate according to ATC and surgical codes. Propensity score matching (PSM) was then performed for the 5ARI and non-5ARI cohorts. The PSM model incorporated demographic characteristics, systemic comorbidities, and laboratory findings into a composite score, and patients in the two groups were matched according to this scoring system. After matching, 181,208 individuals were included in each of the 5ARI and non-5ARI groups. The patient selection process is illustrated in Figure 1.

### Primary Outcome

The primary outcome of this study was the occurrence of OAG after the index date. OAG development was defined using the following criteria: (1) diagnosis of OAG using relevant ICD-10-CM codes; (2) receipt of intraocular pressure/tonometry measurement, optical coherence tomography, or visual field exam according to procedure codes; and (3) confirmation of the OAG diagnosis by an ophthalmologist. All included patients were followed until the first occurrence of the primary outcome, withdrawal from the healthcare insurance system, or the end of the TriNetX database follow-up period, which was December 31, 2024.

### Covariate Adjustment

To more accurately evaluate the relationship between 5ARI exposure and subsequent OAG events, the effects of several covariates were incorporated into the multivariable analysis. These variables included age, race, lifestyle factors, socioeconomic and psychosocial conditions, hypertension, diabetes mellitus, ischemic heart disease, hyperlipidemia, cerebrovascular disease, unspecified dementia, vascular dementia, rheumatoid arthritis, ankylosing spondylitis, and systemic lupus erythematosus. These covariates were identified using relevant demographic information, ICD-10-CM codes, and laboratory codes. To ensure that systemic diseases and demographic factors had persisted long enough to potentially affect the risk of OAG, only comorbidities documented for more than two years were included in the subsequent analyses.

### Statistical Analysis

All statistical analyses were performed using SAS software, version 9.4 (SAS Institute Inc., Cary, NC, USA). Descriptive statistics were used to summarize the baseline characteristics of the 5ARI and non-5ARI groups. The standardized mean difference (SMD) was calculated to assess between-group differences for each baseline variable, and an SMD greater than 0.1 was considered clinically meaningful in this study. Cox proportional hazards regression analysis was conducted to compare the risk of OAG development between the 5ARI and non-5ARI groups. Adjusted hazard ratios (aHRs) and corresponding 95% confidence intervals (CIs) were calculated for OAG incidence. In addition, age, sex, urbanization level, income level, hypertension, diabetes, ischemic heart disease, hyperlipidemia, cerebrovascular disease, unspecified dementia, vascular dementia, rheumatoid arthritis, ankylosing spondylitis, and systemic lupus erythematosus were included in the Cox regression model to control for their potential influence on OAG occurrence. Kaplan-Meier curves were generated to compare the cumulative incidence of OAG between the two study groups, and differences were evaluated using the log-rank test. Additionally, we classified OAG as HTG and NTG via the ICD-10-CM codes, and we also compared the cumulative incidence of HTG and NTG between the two groups. For sensitivity analyses, individuals were stratified into subgroups according to age, race, hypertension, diabetes mellitus, and dyslipidemia. Cox proportional hazards regression was then repeated to estimate the risk of OAG development within each subgroup. A P value less than 0.05 was considered statistically significant, and values below 0.001 were reported as P < 0.001 in this study.

## Results

The baseline characteristics of patients in the 5ARI and non-5ARI cohorts are summarized in Table 1. The average age was 70.8±8.4 years in the 5ARI group and 70.8±8.4 years in the non-5ARI group, with no meaningful difference observed between the two groups (SMD = 0.001). Other demographic variables, including race, socioeconomic and psychosocial factors, and lifestyle-related characteristics, were also comparable between the two cohorts, with SMD values all below 0.1. Similarly, the prevalence of systemic comorbidities was well balanced between the 5ARI and non-5ARI groups, as all corresponding SMD values were less than 0.1 (Table 1).

During the entire follow-up period of up to 10 years, OAG occurred in 3477 patients (5.14%) in the 5ARI group and 3044 patients (4.59%) in the non-5ARI group. After adjustment for multiple covariates, exposure to 5ARIs was associated with a significantly greater risk of OAG compared with non-5ARI treatments (aHR: 1.10, 95% CI: 1.05-1.16, P < 0.001) (Table 2). The Kaplan-Meier analysis comparing OAG occurrence between the two cohorts is presented in Figure 2. The cumulative incidence of OAG was significantly higher among patients receiving 5ARI therapy than among those in the non-5ARI group (P < 0.001) (Figure 2A). In addition, the cumulative incidence of HTG and NTG was significantly higher in the 5ARI population than the non-5ARI cohort (P = 0.002) (Figure 2B), while the cumulative incidence of NTG was similar in both the 5ARI and non-5ARI groups (P = 0.490) (Figure 2C).

In the sensitivity analysis, the 5ARI cohort exhibited significantly increased rates of OAG compared to the non-5ARI population across different subgroups, except for Asian individuals and those without dyslipidemia (Figure 3). Furthermore, the 5ARI cohort demonstrated significantly increased rates of HTG compared with the non-5ARI cohort across different subgroups except those over 70 years of age, Asians, those without hypertension, and those without dyslipidemia (Figure 4). Conversely, among individuals with different features, there was no difference in the NTG occurrence between subgroups (Figure 5).

## Discussion

In previous studies, excess androgen activity has been linked to a variety of disorders involving both reproductive and non-reproductive systems 5, 18. BPH is characterized by increased androgenic influence, urinary incontinence, and difficulty urinating 18, 19. Mild forms of BPH may be treated with androgen deprivation therapy, whereas more advanced cases may require surgical intervention, such as transurethral resection of the prostate 18, 20. In contrast, androgenetic alopecia is primarily associated with elevated androgen activity and progressive hair loss, and it can be managed using minoxidil or anti-androgen therapies such as finasteride 21. Androgen deprivation therapy is also used in the management of prostate cancer 22. However, it can lead to several adverse effects, including skin rashes, fatigue, muscle weakness, and metabolic abnormalities 8. Its use has also been associated with an increased likelihood of sexual dysfunction 6, 8. In addition, non-alcoholic fatty liver disease has been reported in patients receiving androgen deprivation therapy 9, and vascular defects, such as cardiovascular disease, have been reported in individuals receiving 5ARI therapy 23, 24. Regarding the mechanisms of HTG and NTG, HTG (or general OAG) is associated with elevated intraocular pressure, retinal ganglion cells, and optic nerve fiber death, while blood flow autoregulation dysfunction may be involved in the development of HTG 13. NTG also results from intraocular pressure-related retinal ganglion cells and optic nerve fiber death, but additional factors—including ocular perfusion pressure, vascular bed autoregulation, endothelial dysfunction, and oxidative stress—may contribute to its development 14. In relation to vasculature, OAG is not only significantly associated with intraocular pressure but also vascular defects 13, 25. In a previous article, individuals with coronary heart disease were linked to a higher incidence of OAG development 26. Moreover, NTG is influenced by vascular defects, just as the deep macular vasculature was found to be significantly reduced in individuals with NTG in a previous publication 27. Because 5ARIs may impair the vasculature system and cause ischemic changes, which is a predisposing factor for the development of OAG 13, 14, 24, we speculate that their use may correlate to a higher incidence of OAG. Furthermore, a previous study concluded that the application of 5ARIs may be related to later development of dry eye disease, which indicates a possible effect of 5ARIs on the eyes 28. Our research results support this idea, at least to some degree.

In the present study, 5ARI exposure was associated with an increased risk of OAG development. Previous studies assessing OAG have reported that the use of certain pharmacologic agents, including corticosteroids and cholesterol-lowering agents, may be linked to a higher likelihood of OAG occurrence compared with control populations 29. In addition, earlier studies have shown that hormonal components such as estrogen may alter the OAG risk 30. However, only limited research has specifically examined the relationship between 5ARI therapy and the subsequent development of OAG. To the best of our knowledge, the present work is the first to suggest a positive association between 5ARI therapy and OAG onset. Furthermore, in the Cox proportional hazards regression model, we adjusted for multiple potential predisposing factors of OAG, including age, sex, and diabetes, to minimize their confounding effects. In addition, only OAG events documented after the index date were included, which may have helped to establish a temporal sequence between 5ARI exposure and OAG development. Taken together, these findings suggest that 5ARI use may represent an independent risk factor for the occurrence of OAG. The cumulative probability of OAG development was significantly higher in the 5ARI group than the non-5ARI group, which imply a long-term influence of 5ARIs on OAG risk. In contrast, the cumulative probability of HTG, but not NTG, was significantly higher in the 5ARI group. We think the reason for this difference may due to the low individual numbers in the NTG group, in which the percentage of NTG episodes in all OAG cases was only 3.16%. Still, the AHR of NTG was higher than the AHR of HTG, indicating that 5ARIs may still increase the risk of NTG development if adequate numbers of individuals were enrolled.

In the sensitivity analysis, across different age strata and among patients with metabolic disorders, individuals treated with 5ARIs exhibited a significantly greater risk of OAG and HTG than those in the non-5ARI cohort. In previous studies, age (more than 50) has been recognized as an important predisposing factor for OAG development 13, 31, and our findings are generally consistent with this. An earlier meta-analysis indicated that metabolic syndrome-related conditions, including hypertension, dyslipidemia, and insulin resistance, are linked to an increased risk of OAG occurrence 32. Similarly, another review suggested that diabetes mellitus may contribute to the development of OAG 15. The agreement between the results of this study and earlier research further supports the concept that metabolic abnormalities may serve as common risk factors for OAG, not only in the general population but also in specific groups, such as individuals receiving 5ARI therapy. On the contrary, Asian individuals who received 5ARI treatment demonstrated a similar incidence of OAG and HTG compared to Asian participants who did not receive 5ARI treatment. In previous research, Asian race was not a predisposing or protective factor for OAG. Additionally, there was no available pathophysiology explanation for the negative correlation between Asian races and OAG development. Thus, there is a slight conflict between our results and those of previous studies. A possible explanation for this is that the number of individuals in the Asian subgroup was relatively small: there were only 14,634 individuals included in the Asian race subgroup of each cohort. It is possible that the statistical results would change if more individuals were included. Nevertheless, further experimental and clinical research is warranted to determine the mechanism of this association.

From an epidemiological perspective, BPH is highly prevalent, affecting approximately half of men older than 50 4, 33. Similarly, androgenetic alopecia is a common condition, with nearly 80% of men in Western populations reported to experience this dermatologic disorder 6. Given the substantial burden of these two androgen-related diseases, it is estimated that several million individuals receive 5ARI therapy globally 7, 21. On the other hand, OAG is an ophthalmic burden that affects about 2.93 percent of the European population aged 40-80 years old and more than 66 million individuals in the world 13, 31. Furthermore, OAG can lead to irreversible visual impairment and has been estimated as the most common cause of irreversible blindness in the world 34. Because of the high percentage of 5ARI use and OAG in the human population, any relationship between the two factors should be highlighted.

Several limitations of this study should be acknowledged. First, this study employed a database design that relied on diagnostic, laboratory, procedure, and medication codes rather than a direct review of original clinical documents. Therefore, many clinically relevant variables could not be assessed, including imaging findings of BPH, prostate lesion volume, the extent of androgenetic alopecia, results of BPH-related surgeries, treatment adherence among patients with BPH or androgenetic alopecia, therapeutic responses to 5ARIs, possible recurrence of BPH or androgenetic alopecia, detailed information regarding systemic comorbidities, OAG-related symptoms, optical coherence tomography images, visual field test results, treatment response for OAG, and potential OAG recurrence. Secondly, we classified individuals with BPH and androgenetic alopecia as the same population due to the use of 5ARIs. However, the basic pathophysiology of the two diseases is different, and our selection strategy may contribute to analytical inaccuracy. In addition, the retrospective nature of this project could reduce the homogeneity between the two study populations. Despite similar baseline characteristics, some factors—including smoking extent, medication doses, the presence and extent of hypotension, and the presence and extent of high myopia—could not be assessed because of the limited medical records in the TriNetX database. Due to the design of the TriNetX database, we can confirm the time of glaucoma development. However, the details after the glaucoma diagnosis, including the exact type and dosage of glaucoma medications, could not be accessed. This limitation, combined with the absence of visual field tests and optical coherence tomography results, prevented us from performing a glaucoma severity evaluation and could significantly influence the integrity of our conclusions. Finally, we only identified a statistically significant association between 5ARI usage and OAG development, while the exact pathophysiology was not evaluated. The lack of mechanism confirmation could severely reduce the integrity of our results.

In conclusion, the use of 5ARIs may be associated with a higher risk of developing OAG than other non-5ARI treatments. Furthermore, 5ARI therapy may correlate to higher risk of HTG development after adjusting for multiple covariates. Consequently, 5ARIs should be utilized carefully in individuals with known risk factors for OAG development. To evaluate the correlation between 5ARI therapy and OAG outcomes, further prospective, randomized, and large-scale studies are required.

## Figures and Tables

**Figure 1:**
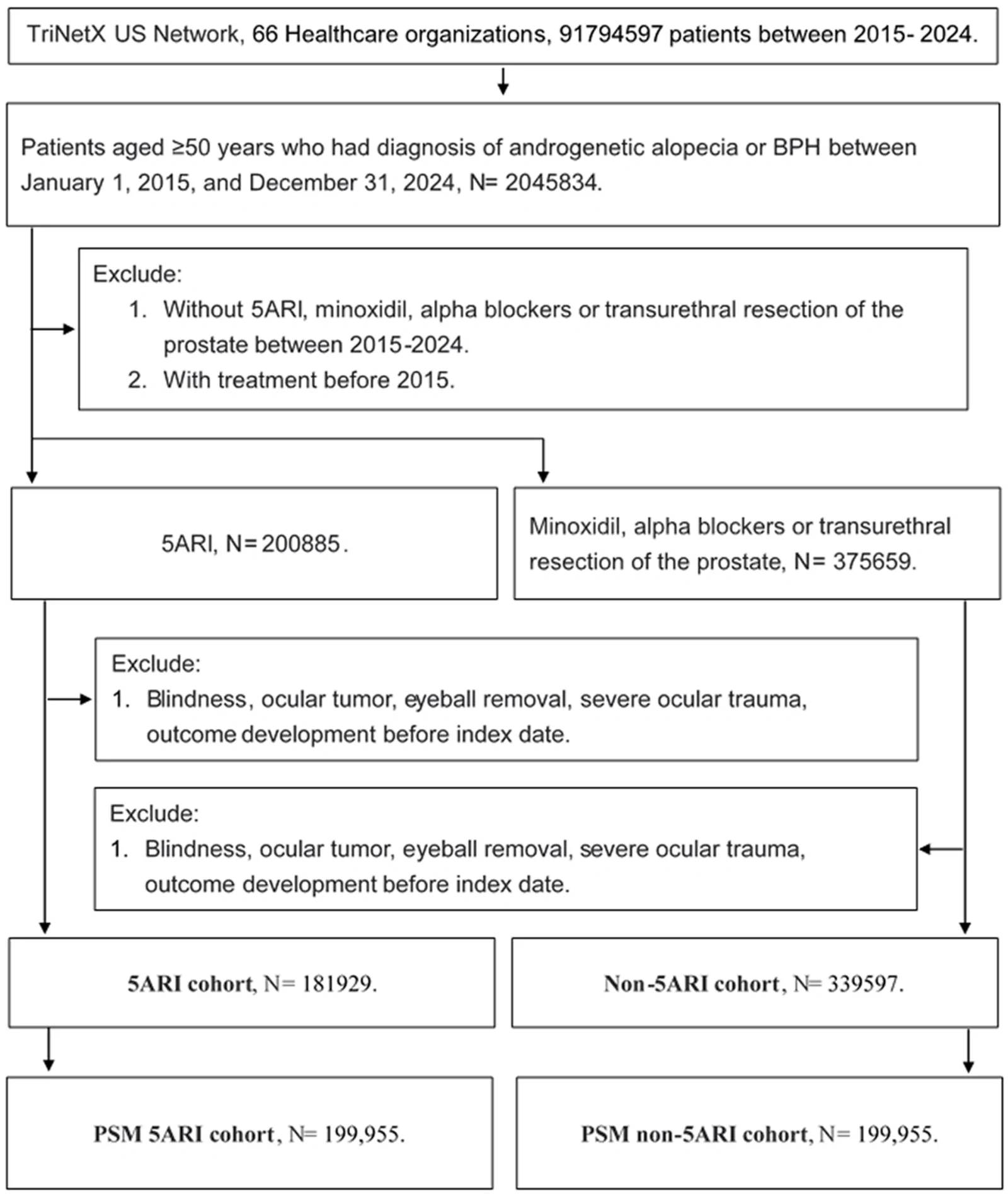
The flowchart of participant selection. 5ARI: 5α-reductase inhibitors, BPH: benign prostate hyperplasia, N: number, PSM: propensity score matching

**Figure 2:**
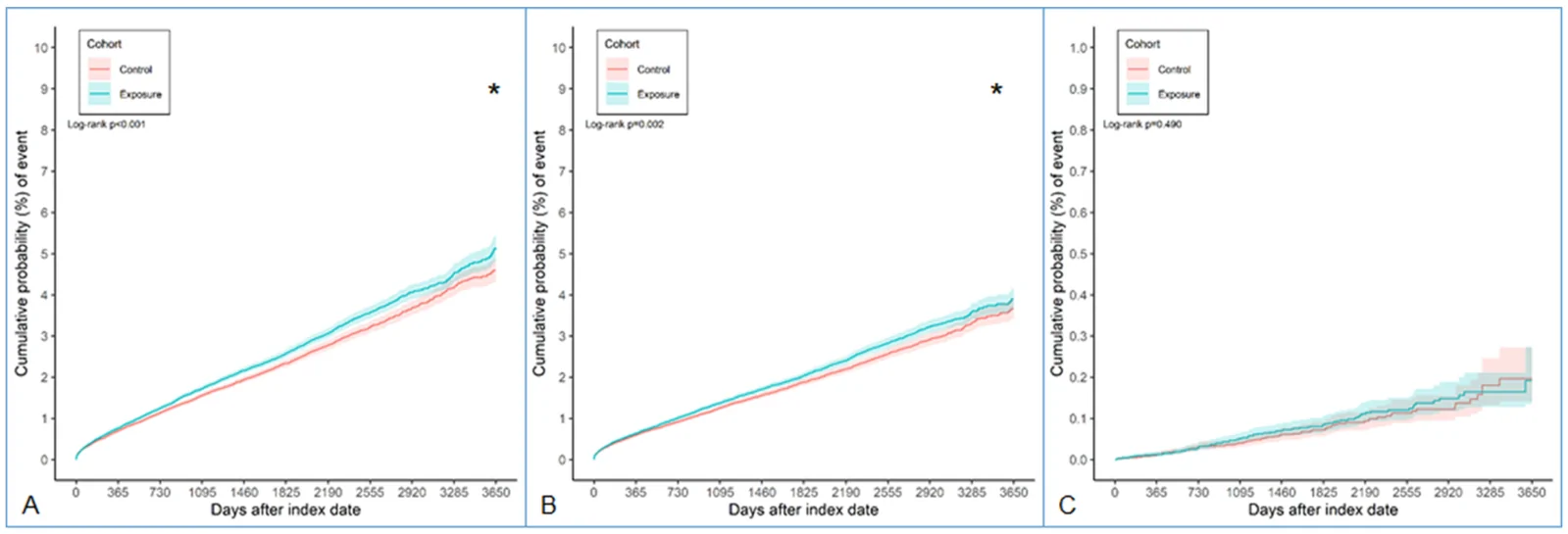
The Kaplan-Meier curve and cumulative incidence of open angle glaucoma between groups. (A) The cumulative incidence of open angle glaucoma. (B) The cumulative incidence of high-tension glaucoma. (C) The cumulative incidence of normal-tension glaucoma. * denotes significant difference between groups

**Figure 3:**
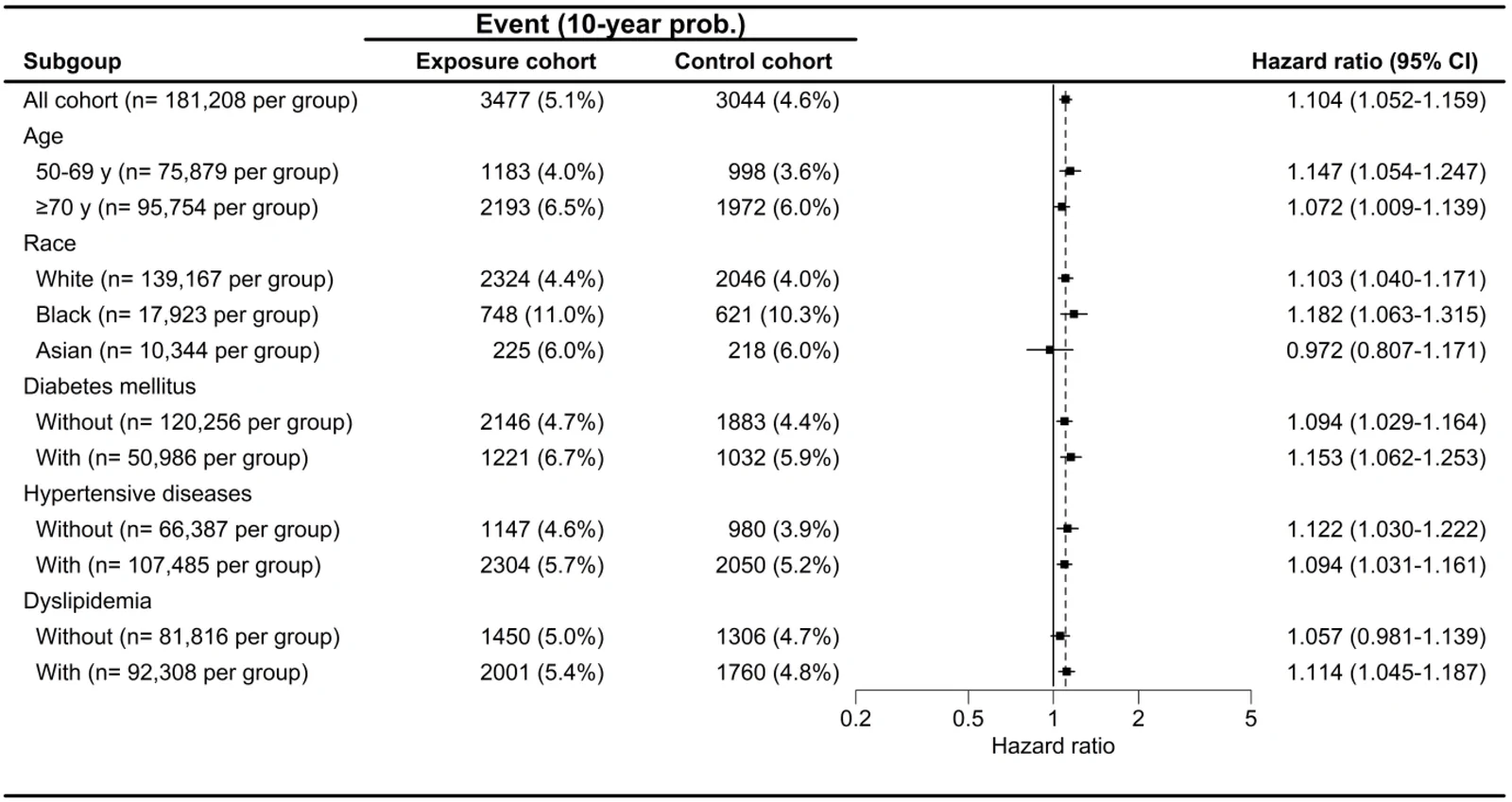
Risk of open angle glaucoma in patients with 5α-reductase inhibitors usage stratified by age, race, diabetes mellitus, hypertension and dyslipidemia. CI: confidence interval, N: number

**Figure 4:**
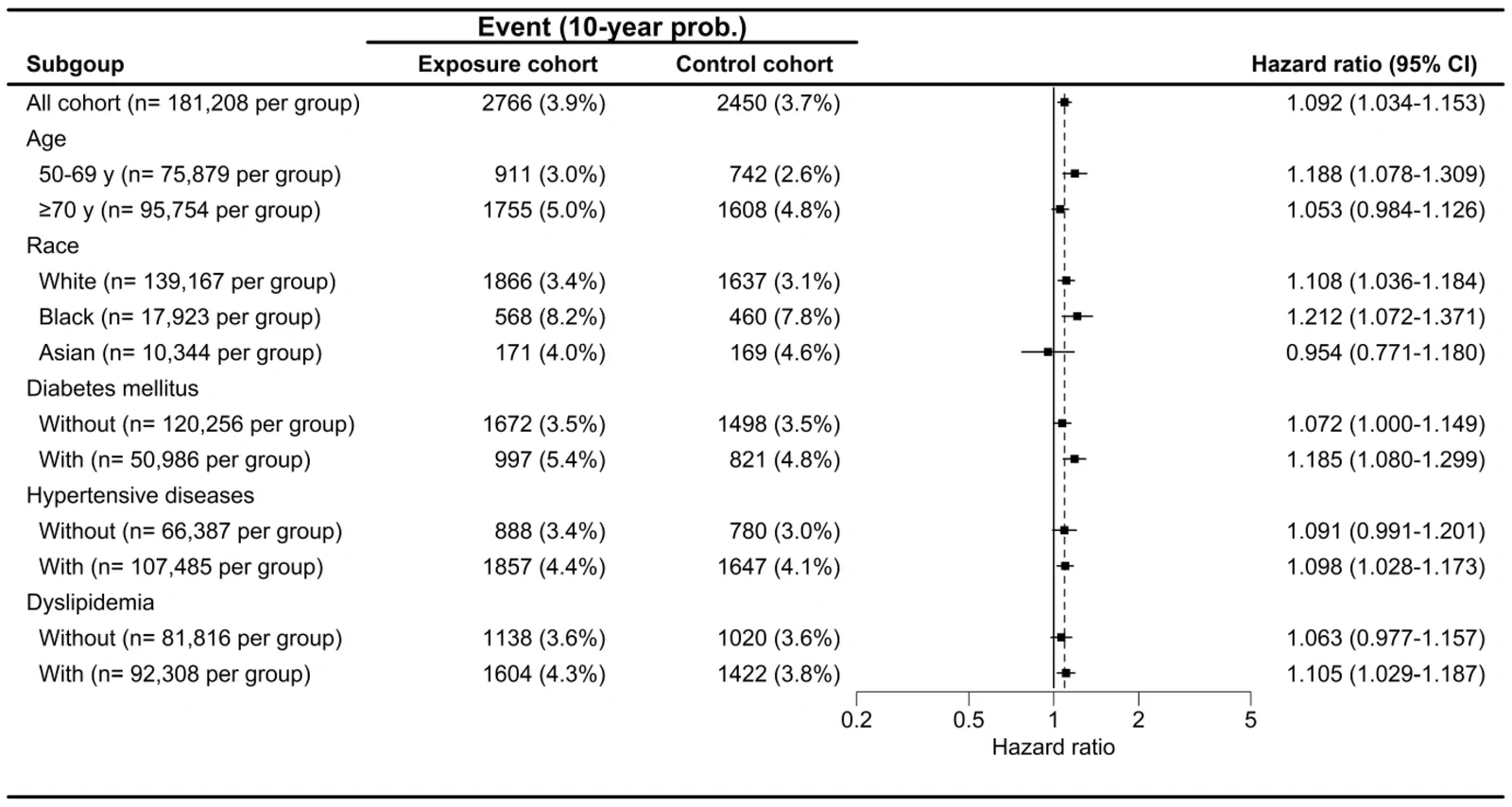
Risk of high-tension glaucoma in patients with 5α-reductase inhibitors usage stratified by age, race, diabetes mellitus, hypertension and dyslipidemia. CI: confidence interval, N: number

**Figure 5:**
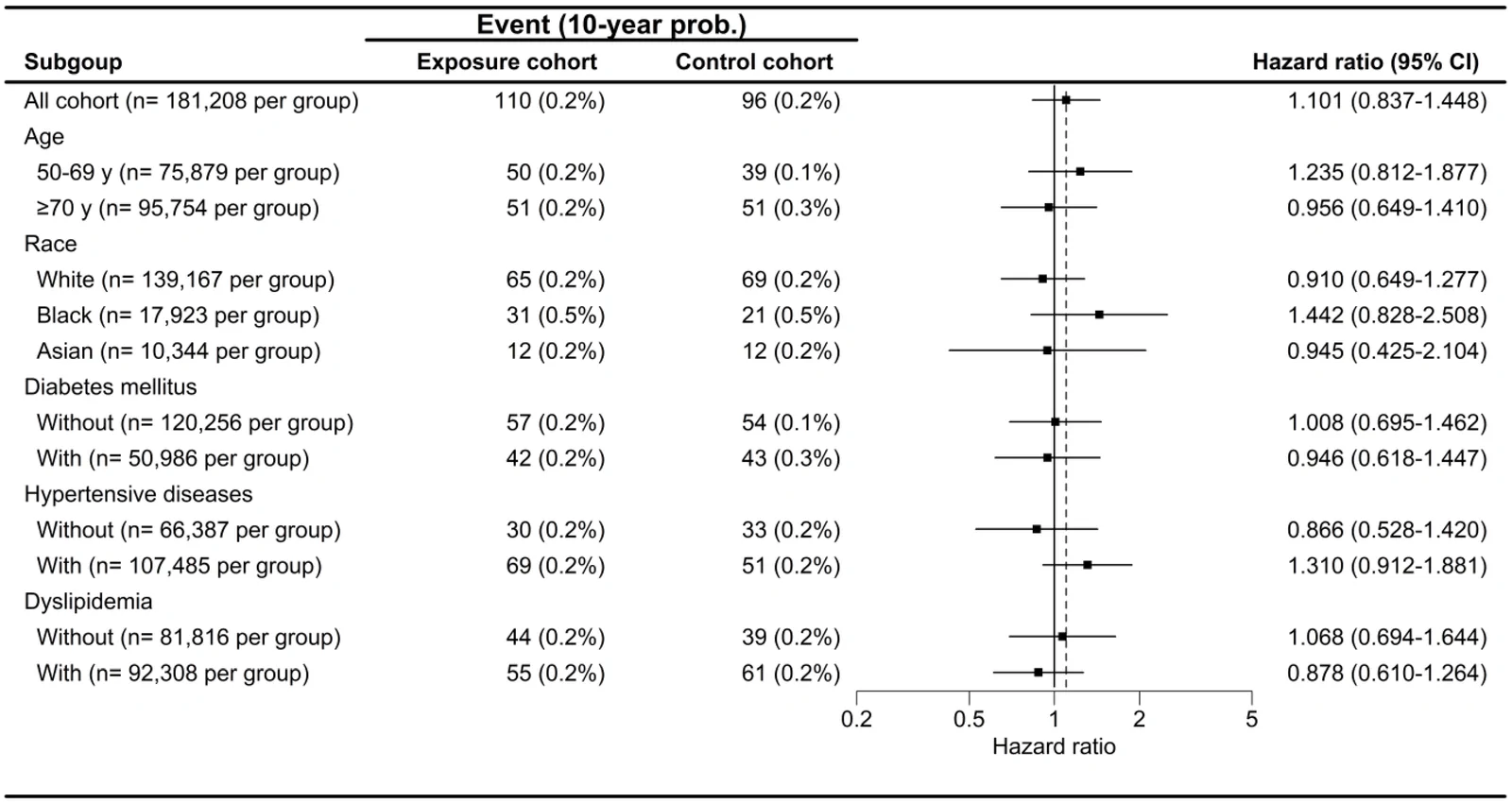
Risk of normal-tension glaucoma in patients with 5α-reductase inhibitors usage stratified by age, race, diabetes mellitus, hypertension and dyslipidemia. CI: confidence interval, N: number

**Table 1:** Baseline characteristics among exposure and control cohort after propensity score matching

Characteristics	5ARI cohort	Non-5ARI cohort	SMD
N	181208	181208	
Age at Index	70.8±8.4	70.8±8.4	0.001
Race			
White	134316(74.1%)	135396(74.7%)	0.014
African American	16744(9.2%)	16189(8.9%)	0.011
Asian	10286(5.7%)	9989(5.5%)	0.007
Psycho-socioeconomic circumstances	3028(1.7%)	2444(1.3%)	0.026
Lifestyle			
Nicotine dependence	15398(8.5%)	14283(7.9%)	0.022
Alcohol related disorders	5316(2.9%)	4533(2.5%)	0.027
Comorbidities			
Hypertensive diseases	106596(58.8%)	106104(58.6%)	0.006
Dyslipidemia	90356(49.9%)	90352(49.9%)	0.000
Diabetes mellitus	47040(26.0%)	46561(25.7%)	0.006
Ischemic heart diseases	45162(24.9%)	44354(24.5%)	0.010
Cerebrovascular diseases	17351(9.6%)	16115(8.9%)	0.024
Dementia	6428(3.5%)	5777(3.2%)	0.020
Nervous degenerative diseases	3312(1.8%)	2881(1.6%)	0.018
Vascular dementia	1303(0.7%)	1096(0.6%)	0.014
Medication			
Nitrofurantoin	3245(1.8%)	2944(1.6%)	0.013
Hydroxychloroquine	934(0.5%)	773(0.4%)	0.013
Phenothiazines	565(0.3%)	462(0.3%)	0.011
Tamoxifen	36(0.0%)	19(0.0%)	0.008

5α-reductase inhibitors: 5ARI, N: number, SMD: standard mean difference

**Table 2:** Open angle glaucoma events in the two groups after follow-up

Study event	N	Cumulative probability			AHR (95% CI)	P
		1-year	5-year	10-years		
OAG						
5AR	3477	0.76 (0.72-0.80)	2.57 (2.48-2.67)	5.14 (4.84-5.47)	1.10 (1.05-1.16)	<0.001*
Non-5ARI	3044	0.70 (0.66-0.74)	2.33 (2.24-2.43)	4.59 (4.32-4.87)	Reference	
HTG						
5AR	2766	0.63 (0.60-0.67)	2.04 (1.95-2.13)	3.90 (3.65-4.15)	1.09 (1.03-1.15)	0.002*
Non-5ARI	2450	0.60 (0.56-0.64)	1.88 (1.79-1.97)	3.65 (3.41-3.91)	Reference	
NTG						
5AR	110	0.01 (0.01-0.02)	0.08 (0.07-0.11)	0.19 (0.13-0.27)	1.10 (0.84-1.45)	0.490
Non-5ARI	96	0.01 (0.01-0.02)	0.07 (0.06-0.09)	0.20 (0.14-0.27)	Reference	

5α-reductase inhibitors: 5ARI, aHR: adjusted hazard ratio, CI: confidence interval, HTG: high-tension glaucoma, N: number, NTG: normal-tension glaucoma, OAG: open angle glaucoma. * denotes significant difference between groups

## Abbreviations

5ARI: 5α-reductase inhibitor
aHR: adjusted hazard ratio
BPH: benign prostate hyperplasia
CI: confidence interval
eGFR: estimated glomerular filtration rate
HTG: high-tension glaucoma
ICD-10: International Classification of Diseases Tenth Revision
N: number
NTG: normal-tension glaucoma
OAG: open-angle glaucoma
PSM: propensity score matching
SMD: standard mean difference
